# Immuno-modulatory role of baicalin in atherosclerosis prevention and treatment: current scenario and future directions

**DOI:** 10.3389/fimmu.2024.1377470

**Published:** 2024-04-18

**Authors:** Li Wang, Shenyi Huang, Xiaolun Liang, Junliang Zhou, Yifan Han, Jiangshan He, Danping Xu

**Affiliations:** Department of Traditional Chinese Medicine, The Eighth Affiliated Hospital of Sun Yat-Sen University, Shenzhen, China

**Keywords:** baicalin, atherosclerosis, immuno-modulatory, traditional Chinese medicine, anti-inflammatory

## Abstract

Atherosclerosis (AS) is recognized as a chronic inflammatory condition characterized by the accumulation of lipids and inflammatory cells within the damaged walls of arterial vessels. It is a significant independent risk factor for ischemic cardiovascular disease, ischemic stroke, and peripheral arterial disease. Despite the availability of current treatments such as statins, proprotein convertase subtilisin/kexin type 9 (PCSK9) inhibitors, and lifestyle modifications for prevention, AS remains a leading cause of morbidity and economic burden worldwide. Thus, there is a pressing need for the development of new supplementary and alternative therapies or medications. Huangqin (*Scutellaria baicalensis* Georgi. [SBG]), a traditional Chinese medicine, exerts a significant immunomodulatory effect in AS prevention and treatment, with baicalin being identified as one of the primary active ingredients of traditional Chinese medicine. Baicalin offers a broad spectrum of pharmacological activities, including the regulation of immune balance, antioxidant and anti-inflammatory effects, and improvement of lipid metabolism dysregulation. Consequently, it exerts beneficial effects in both AS onset and progression. This review provides an overview of the immunomodulatory properties and mechanisms by which baicalin aids in AS prevention and treatment, highlighting its potential as a clinical translational therapy.

## 1 Introduction

Atherosclerosis (AS) is a chronic and progressive disease characterized by inflammation within the arterial wall, accompanied by the accumulation of overactive smooth muscle cells and immune cells. This condition forms the pathophysiological basis for cardiovascular disease (CVD) and ischemic stroke, leading to significant mortality and disability worldwide (1–4). Although recent advancements in revascularization techniques and cholesterol-lowering medications (primarily statins and PCSK9 inhibitors) have notably improved AS management (5–7), the incidence of AS-induced acute events, such as acute myocardial infarction and acute ischemic stroke, continues to be alarmingly high over the past decade, contributing to substantial economic burden and mortality rates in both developed and developing countries (8). For instance, in China and many Asian countries, the number of newly diagnosed AS cases has been continuously increasing, and AS-associated cardiovascular disease or thrombosis remains a leading cause of death, which is reported to be as high as 40% (9–11). In light of these challenges, there is a consensus on the necessity for complementary and alternative treatment approaches for AS. Current guidelines strongly recommend pharmacological interventions, yet the effectiveness of traditional Chinese medicine (TCM) has garnered interest from the AS and CVD research community because of its “multiple channels and multiple targets” approach (12–14). Significantly, TCM has a higher acceptance in China and other Asian countries, owing to its strong cultural affinity. Therefore, developing TCM-based therapies for AS prevention and treatment represents a promising direction for novel strategic interventions.

Baicalin, a prominent flavonoid compound extracted from the root of Huangqin (*Scutellaria baicalensis* Georgi. [SBG]), exhibits a wide range of pharmacological effects such as anti-inflammatory, anti-apoptotic, antioxidant, and anticancer properties as well as improvement of smooth muscle cell function (15–19). In line with these properties, baicalin presents promising opportunities for AS prevention and treatment, as its therapeutic mechanisms and functional targets are likely linked to the pathophysiological processes of AS (20–22). This review draws upon the pathological basis of AS and provides a comprehensive analysis of the latest research on the anti-atherosclerotic effects of baicalin from an immunomodulatory perspective, aiming to offer insights into future directions for developing innovative treatment strategies for AS.

## 2 Immuno-interaction along with the processes in AS

**Figure 1:**
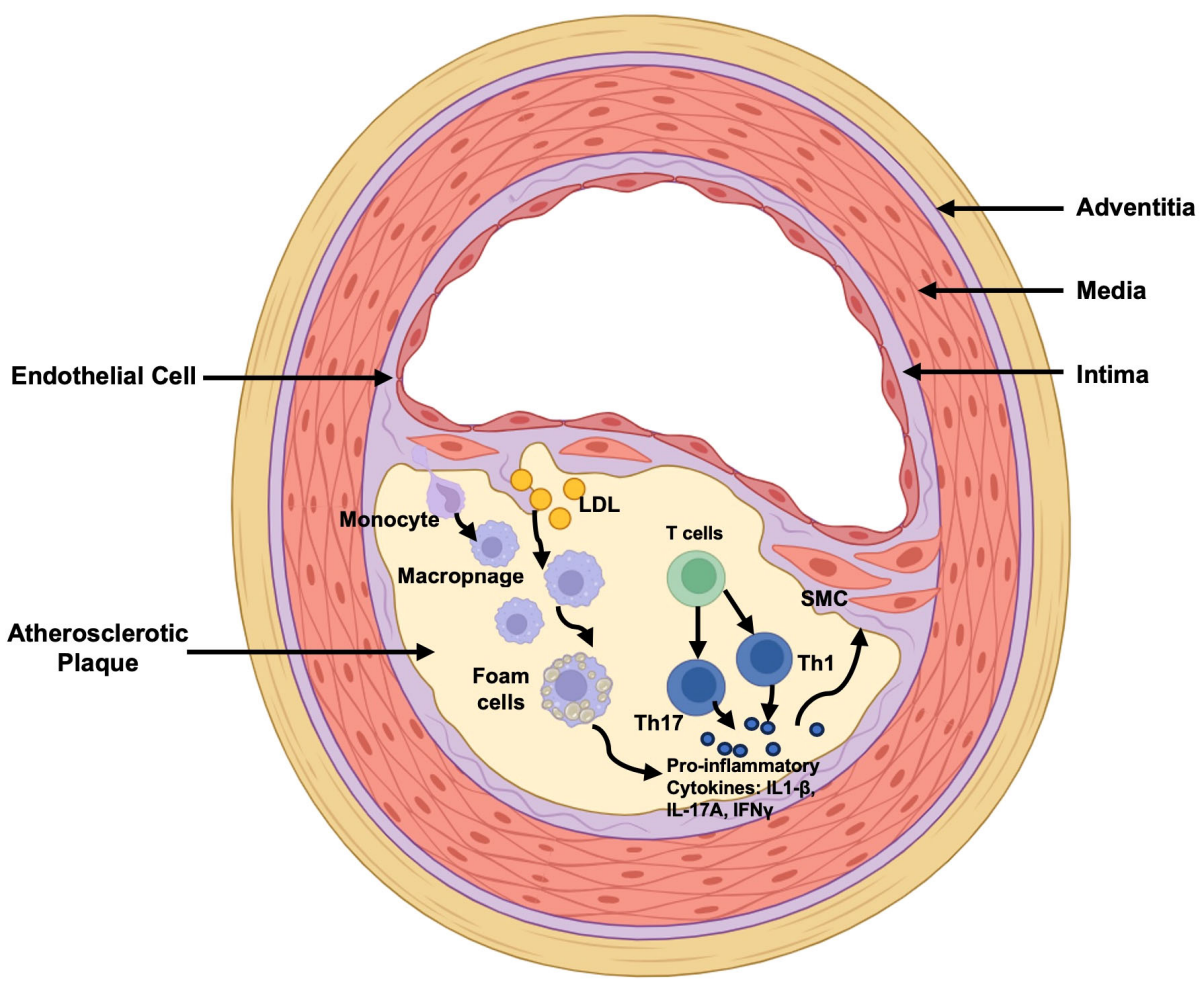
Main Immuno-alteration in Atherosclerosis. LDL retention is the marker of the initiation of atherosclerosis. Monocytes transmigrate into the subendothelial space and differentiate into macrophages, following binding to lipoproteins and forming foam cells which secret pro-inflammatory cytokines IL-1β for down-streaming smooth muscle cell activation for further pro-atherosclerotic processes. In the meantime, T cells are differentiated into Th1 and Th17 cells to secret IFNγ and IL-17A exerting pro-inflammatory effects. LDL, Low-Density Lipoprotein; Th17, T helper cells 17; Th1, T helper cells 1; IL-1β, interleukin (IL)-1β; IL-17A, interleukin (IL)-17A; IFNγ, Interferon-gamma; SMC, Smooth Muscle Cell.

Immune system plays a crucial role in the initiation and progression of AS. Here, we will shortly introduce the main involvements of innate and adaptive immunity during AS pathophysiological processes (**Figure 1**).

### 2.1 Innate immunity alteration in AS

In the initial stages of AS, endothelial cell damage triggered by various inflammatory responses and ischemic-hypoxia events leads to the release of adhesion and chemotactic cytokines. These cytokines attract monocytes and other leukocytes to the site of inflammation, where they mature and transition into macrophages (23–25). The intracellular accumulation of cholesterol in macrophages activates surface scavenger receptors, facilitating the binding of macrophages to lipoprotein particles and leading to foam cell formation (26, 27). Additionally, lipid accumulation prompts endothelial cells to secrete inflammatory mediators and cytokines, further accelerating AS onset. Additionally, dendritic cells (DCs) accumulating in atherosclerotic plaques play a crucial role in the disease’s progression by presenting oxidized low-density lipoprotein (ox-LDL)-derived antigens, which activate both humoral and adaptive immune responses (28, 29).

### 2.2 Adaptive immunity alteration in AS

In the advanced stages of AS, adaptive immunity, especially cellular immunity, plays a role in T-cell infiltration (**Figure 1**) (30, 31). Although T cells are not as abundant as monocytes, which undergo differentiation into macrophages, they can penetrate the intima to modulate the functions of innate immune cells and smooth muscle cells. T helper cells 1 (Th1) exert a pro-inflammatory role in AS, for example, by secreting interferon-gamma (IFN-γ), which promotes monocyte infiltration, macrophage differentiation, and foam cell formation. T helper cells 2 (Th2), on the other hand, by secreting interleukin (IL)-4 to play a disease prompting role. T helper cells 17 (Th17), activated under pro-atherogenic conditions, have complex effects because of the various roles of IL-17A at different AS stages. However, undoubtedly, the proportion of Th17 cells increases in patients with acute coronary syndrome. Conversely, regulatory T (Treg) cells are present in atherosclerotic plaques at all stages of AS, although in a smaller proportion (32, 33). Animal studies have reported the exacerbating effects of depleting Tregs in AS, while reintroducing Treg cells mitigates and slows disease progression (34). Mechanistically, Treg cells secrete IL-10, playing a crucial role in preventing AS progression. Additionally, Treg cells secrete transforming growth factor-beta (TGF-β) to inhibit inflammation and the proliferation of smooth muscle cells. While the role of humoral immunity in AS development is little known to us since B cells are occasionally detected in atherosclerotic plaques. Studies on B-cell dynamics in atherosclerotic mice have yielded contradictory results: one indicates B cells mitigate AS, while another suggests B cells exacerbate it following B cell depletion (35–38). This discrepancy underscores the need for a comprehensive examination of B cell populations and their specific functions.

Collectively, these findings indicate that the pathological mechanisms of AS involve the participation of endothelial cells, monocyte-derived macrophages, smooth muscle cells, and immune cells. Consequently, understanding the role of immune interactions during AS progression could pave the way for innovative strategies targeting these immune responses. Furthermore, baicalin can modulate immune cells and their actions in AS progression, positioning it as a promising approach for AS prevention and treatment.

## 3 Immuno-modulatory features of baicalin in AS

**Figure 2:**
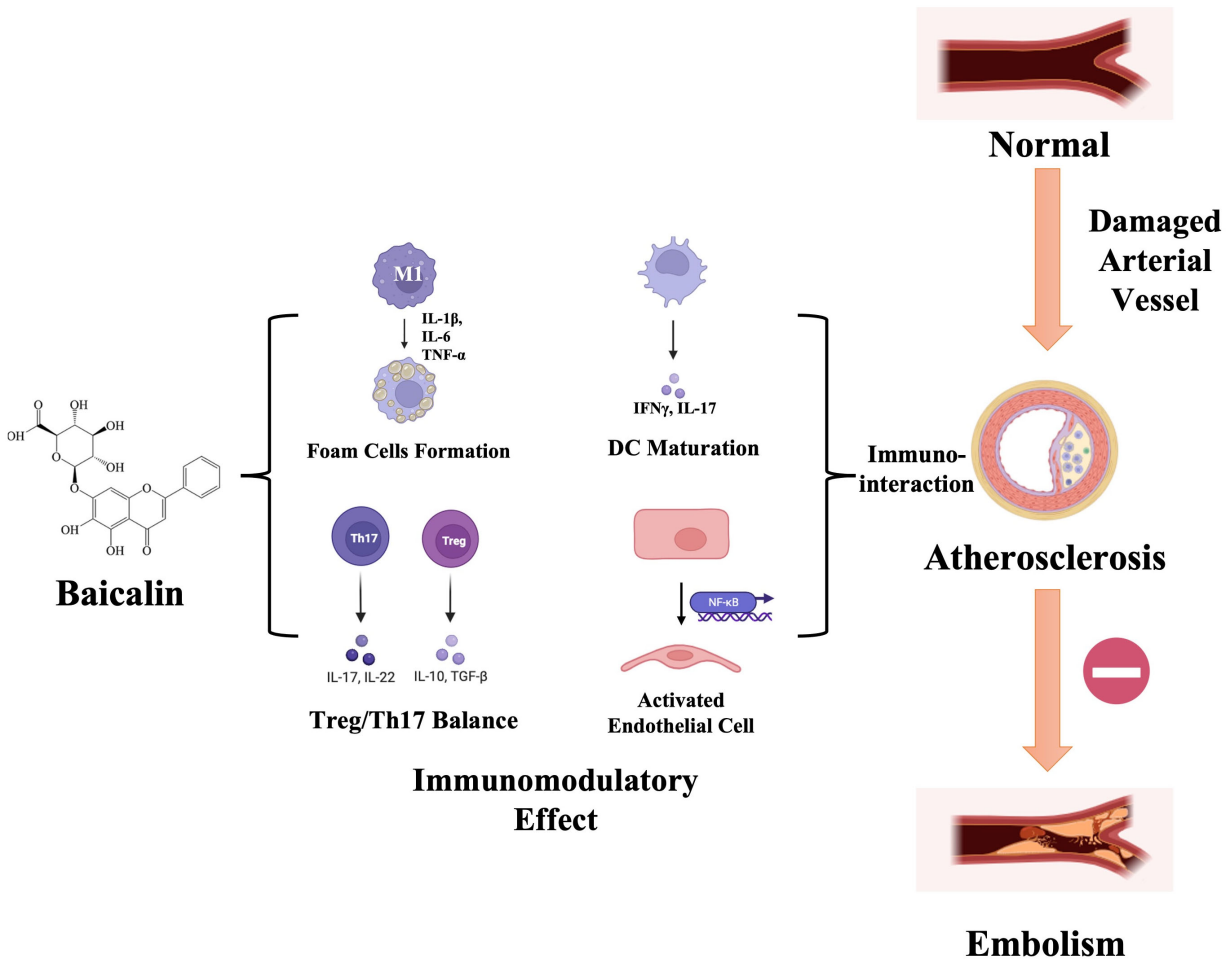
Schematic illustration of baicalin’s immunomodulatory effects during atherosclerosis progression. Upon damage manifestation within arterial vessels, diverse immune cell populations undergo activation and subsequent migration toward the intima, thereby instigating the development of an inflammatory microenvironment. Baicalin, with its immunomodulatory ability, specifically targets the differentiation of macrophage-foam cells, imbalance between Treg/Th17 cells, dendritic cell (DC) maturation, and modulation of inflammatory signaling pathways. This orchestrated modulation serves a dual purpose of impeding the progression of atherosclerosis toward embolism and mitigating the risk of acute ischemic events. IL-17, interleukin (IL)-17; IFNγ, Interferon-gamma; DC, Dendritic Cell; NF-κB, Nuclear Factor-kappa B; IL-1β, interleukin (IL)-1β; IL-6, interleukin (IL)-6; TNF-α, Tumor Necrosis Factor-α; IL-22, interleukin (IL)-22; IL-10, interleukin (IL)-10; TGF-β, Transforming Growth Factor Beta; Th17, T helper cells 17; Treg, regulatory T cells.

Extensive research has confirmed baicalin’s multifaceted pharmacological benefits, including anti-apoptotic effects, enhancement of endothelial cell functions, antioxidant properties, and modulation of host immune homeostasis. These attributes contribute to its protective role in both the initiation and progression of AS (**Figure 2**). This review focuses on the immunomodulatory effects of baicalin in AS prevention and treatment, underscoring its potential as a significant therapeutic agent.

### 3.1 Effect of baicalin on innate immunity in AS

After migrating to the intima, monocytes undergo differentiation into macrophages. These macrophages then engulf large amounts of ox-LDL through scavenger receptors, leading to the formation of foam cells (26). These foam cells play a crucial role in the progression of AS lesions, making the transition of macrophages into foam cells a critical phase in AS development and progression. Consequently, strategies aimed at promoting macrophage apoptosis and preventing of macrophage foam cell formation have emerged as key focus areas in AS prevention and treatment. Baicalin primarily functions by inhibiting macrophage foam cell formation, regulating cholesterol homeostasis, and facilitating macrophage apoptosis. Baicalin triggers macrophage apoptosis by activating three signaling pathways, namely c-Jun N-terminal kinases (JNKs), p38 mitogen activated protein kinase (p38/MAPK), and Steroid Receptor RNA Activator (SRA) (39), in response to lipopolysaccharide (LPS) and acetylated low-density lipoprotein (Ac-LDL), thus preventing foam cell formation in diseased cells. In studies utilizing an LPS-sensitized mouse macrophage model, baicalin demonstrated potential in modulating Protein kinase A (PKA) activity in macrophages, which, in turn, may suppress NLR family pyrin domain containing 3 (NLRP3) inflammasome activation and cell pyroptosis (40). Additionally, Baicalin encourages M2 macrophage polarization and enhances phagocytosis by increasing Mer Tyrosine Kinase (MERTK) receptor expression (41).

Macrophages in AS primarily display two phenotypes: M1 and M2 types. In general, M1 macrophages, also known as classically activated macrophages, are typically involved in the initial pro-inflammatory response. In contrast, M2 macrophages contribute to anti-inflammatory processes and produce anti-inflammatory cytokines. In experiments investigating the effects of baicalin on RAW 264.7 macrophages cell line, it was found that Baicalin inhibited the expression of inflammatory factors IL-1β, IL-6, Tumor Necrosis Factor-α (TNF-α), Cyclooxygenase-2 (COX-2) in macrophage-derived foam cells. This upregulation promoted the expression of immunosuppressive factors TGF-β and IL-10, leading to the polarization of macrophages from M1 to M2 type, which inhibited the inflammatory response during the AS process. Further analysis, combined with relevant literature, revealed a potential association between this mechanism and Baicalin’s activation of the key protein peroxisome proliferator-activated receptor gamma (PPAR-γ) (42, 43). Previous studies have shown that Baicalin exhibits anti-inflammatory activity by inhibiting the activation of the Toll-like Receptor 4 (TLR4)/Myeloid Differentiation primary response 88 (MyD88)/Nuclear Factor-kappa B (NF-κB) cell signaling pathway (44). Recent research has further confirmed that Baicalin inhibits inducible Nitric Oxide Synthase (iNOS) gene functions, providing additional insight into Baicalin’s anti-inflammatory effects in AS (45). Baicalin can also inhibit macrophage proliferation, suppress excessive production of pro-inflammatory cytokines triggered by S100A8/A9, and reduce expression of the NADPH oxidase 2 (NOX2) gene, thereby inhibiting the inflammatory response (46). Despite the increasing understanding of the mechanisms by which Baicalin inhibits macrophage foam formation, further exploration is necessary to investigate its mechanisms for anti-foam cell formation and cholesterol metabolism from multiple perspectives. Additionally, compelling evidence shows that baicalin can reduce CD11c expression on DCs within atherosclerotic plaques, thus inhibiting DC maturation and the development of atherosclerotic lesions (47–49). Baicalin may also promote the transition of conventional DCs into plasmacytoid DCs and alter the expression of functional molecules by blocking the Signal Transducer and Activator of Transcription 5-Inhibitor of DNA binding 2 (STAT5-ID2) pathway, further demonstrating its protective effect against AS (50).

### 3.2 Effect of baicalin on cellular adaptive immunity in AS

Adaptive immunity plays a crucial role in both the onset and progression of AS (23). Activated T cells, which have encountered antigens before, are present within atherosclerotic plaques. Studies using immunodeficient apo E ^−/−^ animal models, which lack both T cells and B cells, have shown fewer atherosclerotic events compared with their immunocompetent counterparts (51, 52). This evidence underscores the pro-atherosclerotic influence of T cells and suggests that immunosuppressive strategies may be beneficial for treating AS. Treg cells, in particular, contribute to the protection against AS development by releasing anti-inflammatory cytokines such as IL-10 and TGF-β. Supporting this notion, baicalin can enhance Forkhead Box P3 (Foxp3) expression, thereby promoting the differentiation of Treg cells within atherosclerotic plaques, and exerting anti-arteriosclerotic effects (22, 53, 54). The underlying mechanism is believed to involve baicalin’s inhibition of the Mammalian Target of Rapamycin (mTOR) pathway, leading to downregulated expression of Ras homolog gene family, member A (RhoA), Rho-Associated Coiled-Coil-Containing Protein Kinase 1 (ROCK1), phosphorylated STAT4 (p-STAT4), and p-STAT3, while simultaneously increasing phosphorylated STAT5 expression. The latter directly interacts with the Foxp3 gene, enhancing Treg cell function. Additionally, baicalin can influence the gut microbiome in an ulcerative colitis animal model, balancing Treg/Th17 ratios (55) and exhibiting anti-inflammatory properties. This suggests potential for further research into baicalin’s effects on the microbiome in AS. Baicalin also inhibits Th17 cell differentiation by downregulating IL-6 and Retinoic Acid Receptor-Related Orphan Receptor Gamma (RORctγ) expression (56). Yet, its effect on the Th1 cell population, which secretes IFN-γ and contributes to AS progression, remains less explored, warranting further investigation. Overall, the evidence supports baicalin’s immunomodulatory benefits in AS, particularly regarding Treg/Th17 balance.

### 3.3 Effect of baicalin on humoral adaptive immunity in AS

Baicalin’s effect extends beyond adaptive immune cells, also influencing humoral immunity. However, the relationship between humoral immunity and AS presents conflicting evidence, necessitating further research (23). Baicalin is reported to significantly boost B cell proliferation and IL-12 production through its interaction with TLR4 (57). Additionally, by modulating signaling pathways such as NF-κB or Phosphoinositide 3-kinase/Protein Kinase B (PI3K/Akt), which are critical for B cell survival and homeostasis, Baicalin can potentially influence the lifespan and turnover of B cells which eventually affecting the quantity and quality of antibodies produced by B cells. The interplay between innate and adaptive immunity in the onset and progression of AS highlights the potential of baicalin as a therapeutic agent, primarily through its ability to regulate immune homeostasis.

### 3.4 Effect of baicalin on inflammation responses in AS

Inflammation is a critical driver at all stages of AS, with chemokines, pro-inflammatory cytokines, adhesion molecules, and inflammatory signaling pathways (5) playing pivotal roles. The NF-κB signaling pathway, in particular, promotes AS by increasing the production of pro-inflammatory cytokines and chemokines such as IL-6, IL-1, Intercellular Adhesion Molecule-1 (ICAM-1), Vascular Cell Adhesion Molecule-1 (VCAM-1), and TNF-α. Therefore, inhibiting NF-κB activation is effective in reducing AS incidence. Baicalin can suppress cytokine and chemokine production by inhibiting NF-κB activation in both *in vitro* and *in vivo* studies. Baicalin pretreatment to human umbilical vein endothelial cells confers protection from damage induced by vascular inflammation, which is similar to the early stages of AS, by suppressing NF-κB activity (58, 59). Baicalin can enhance SIRT expression in models of cardiovascular AS, acting as an inhibitor of NF-κB. This suppression of NF-κB leads to a reduction in the secretion and activation of downstream inflammatory factors (60). Additionally, baicalin influences the Wnt pathway, a crucial cellular signaling network involved in numerous biological processes, playing a significant anti-atherosclerotic role. Baicalin also promotes the activation of the Wingless-related integration site/Dickkopf-1 (Wnt/DKK1) pathway, potentially mitigating AS progression (61). Baicalin can also decrease the levels of inflammatory factors by promoting the phosphorylation of glycogen synthase kinase 3β (GSK3β), enhancing β-catenin expression and nuclear translocation, which is associated with the Wnt pathway (62). In summary, these findings underscore baicalin’s role in attenuating inflammatory responses, thereby decreasing the degree of AS progression.

## 4 Future directions of baicalin in AS

Baicalin has shown potential as a novel therapeutic agent for various diseases, but its clinical development and application (63) have been significantly hindered by its low bioavailability. To address this challenge, several innovative strategies, such as nanonization, solid dispersion, inclusion complexes, and micelles, have been designed and developed. These modifications have been proven to enhance the dissolution and solubility of baicalin. For instance, the formation of a baicalin-phospholipid complex through weak interactions under specific conditions can further improve the bioactivity of baicalin, offering enhanced anti-inflammatory effects with faster onset and prolonged duration (64). Furthermore, the advent of various baicalin-loaded liposome technologies, including folate-modified baicalin-loaded liposomes, baicalin-phospholipid nanoparticles, and baicalin-phospholipid prodrugs, has improved baicalin bioavailability in oral drug delivery systems (65). However, the exploration of baicalin’s drug delivery systems in the context of AS therapies remains limited, underscoring the need for further evaluation of suitable delivery vehicles to leverage its promising anti-atherosclerotic effects.

Recent evidence has highlighted the anti-inflammatory effects of mesenchymal stem cells (MSCs) in treating multiple diseases, including AS (66, 67). Intriguingly, baicalin enhances the proliferation and survival of MSCs by modulating the hedgehog signaling pathway (68), addressing the critical challenge of MSC apoptosis in clinical applications. Moreover, baicalin pretreatment can optimize the therapeutic effects of MSC-derived exosomes by inhibiting the nuclear factor erythroid 2–related factor 2 (Nrf2) pathway (69). These findings suggest that a combined approach of baicalin and MSC therapy could offer superior therapeutic benefits over using either treatment alone. This synergy underscores the potential of developing combination strategies that integrate baicalin and MSCs, warranting further investigation.

## 5 Concluding remarks

Baicalin has been recognized as a promising therapeutic agent for AS prevention and treatment owing to its ability to mitigate inflammation-induced damage, regulate lipid metabolism, promote cell survival, and modulate immune responses. This discussion has focused on the immunomodulatory effects of baicalin in combating AS. Despite extensive experimental evidence supporting baicalin’s immunomodulatory benefits in AS, the specific mechanisms of action, including its precise receptors and downstream signaling pathways, remain elusive. This gap in knowledge, coupled with limited clinical data, has delayed baicalin’s potential clinical applications. Moreover, the physical properties of baicalin still require optimization and technological advancements. Looking forward, initiating appropriate baicalin-based clinical trials is recommended to gather sufficient data, which could enrich our understanding of its mechanisms and enhance its therapeutic potential in AS prevention and treatment.

## Author contributions

LW: Funding acquisition, Writing – original draft, Writing – review & editing. SH: Writing – original draft, Writing – review & editing. XL: Resources, Visualization, Writing – original draft. JZ: Investigation, Writing – review & editing. YH: Resources, Writing – review & editing. JH: Visualization, Writing – review & editing. DX: Supervision, Writing – review & editing.
